# The impact of surgical training on early and long-term outcomes after isolated aortic valve surgery

**DOI:** 10.1093/ejcts/ezab328

**Published:** 2021-08-06

**Authors:** Arnaldo Dimagli, Shubhra Sinha, Umberto Benedetto, Massimo Caputo, Gianni D Angelini

**Affiliations:** Bristol Heart Institute, University of Bristol, Bristol, UK

**Keywords:** Surgical training, Medical education, Aortic valve replacement, Surgical education

## Abstract

**OBJECTIVES:**

Patients presenting with more comorbidities, requiring more complex cardiac surgical procedures and an increase in public scrutiny are impacting on training programme because of the perceived risk of worse outcomes. Hence, we aimed to provide evidence that trainees as the first operator can achieve comparable results to consultants when performing isolated surgical aortic valve replacement.

**METHODS:**

From 1996 to 2017, 2919 patients underwent surgical aortic valve replacement at the Bristol Heart Institute, operated on by either a consultant (*n* = 2220) or a trainee (*n* = 870) as the first operator. Propensity score matching was used to adjust for imbalance in the baseline characteristics of the 2 groups.

**RESULTS:**

Over a 21-year period, the proportion of trainee cases dropped from 41.5% to 25.9%. No differences in the rates and risk of in-hospital mortality, new cerebrovascular accidents, re-exploration for bleeding, deep sternal wound infection and length of stay were found between patients operated on in the 2 groups. Also, there was a comparable risk of late death between the 2 groups (HR 0.88; 95% CI 0.73–1.06; *P* = 0.27) and this was present regardless of trainees career level and patients surgical risk based on the EuroSCORE. Finally, we showed an increase in patients risk profile in the latest year but, this was not associated with the worst outcomes when trainees performed the operation.

**CONCLUSIONS:**

Surgical aortic valve replacement is a safe and reproducible technique and regardless of the patient’s risk profile, and no differences in the outcomes between trainees and consultant cases were found.

**Figure:**
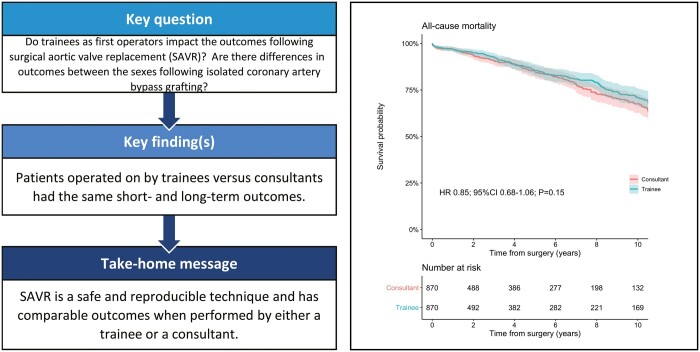

## INTRODUCTION

Over the few past years, research in surgical education has bloomed and attention has been drawn to the quality and quantity of surgical training. The involvement of trainees in the operating theatres is of utmost importance for the development of competent, technically proficient and practice-ready surgeons. In this context, an optimal balance between patients’ safety and a proper surgical exposure exists and training must be provided within a strict framework of patients’ safety. Traditionally, cardiothoracic trainees are involved in cases requiring low-risk and low-complexity procedures in which there is plenty of teaching opportunities. However, in recent years, there has been a noticeable change in the cardiac surgical cohorts, with patients presenting with more comorbidities, and requiring more complex procedures [1–3]. In these cases, the surgical opportunity and responsibilities of trainees may be limited because of the perceived increased risk of possible complications. Moreover, the last decades have seen an increase in the public scrutiny of cardiac surgery outcomes to provide patients with information on hospitals and surgeons performance [4–6]. Thus, consultants may guard their performance outcomes and opt for reducing trainee autonomy in decision-making and operative procedures.

The purpose of this study was to provide evidence regarding the clinical short-term and long-term outcomes after isolated surgical aortic valve replacement (SAVR) performed by trainees as compared to consultants.

## METHODS

### Ethical statement

Ethical and legal requirements were met, and Clinical Audit Committee of the University Hospitals Bristol National Health Service Foundation Trust approved the study and a waiver for patients’ consent was obtained (CARDS/SE/2020–21/04). This study was a retrospective analysis of prospectively collected data from the National Institute for Cardiovascular Outcomes Research (NICOR) registry. We included patients undergoing elective isolated SAVR, at the Bristol Heart Institute, from April 1996 to December 2017.

### Study population

Adult patients were included in the study if they underwent isolated SAVR performed by either a consultant or a trainee supervised by a consultant surgeon. Patients were excluded if they underwent SAVR combined with other concomitant procedures (i.e. coronary artery bypass grafting, other valvular procedures), if they had had previous heart surgery or underwent emergency or salvage procedures.

A procedure performed by trainee as the first operator was defined as a case in which the cardiothoracic trainee performed the entire surgical procedure (‘skin-to-skin’). This operation could be either supervised by a scrubbed consultant acting as first assistant or unsupervised when the consultant was not scrubbed in and trainee reviewed the case and planned the surgical strategy independently. The decision to have a trainee case was at the discretion of individual consultant surgeons.

### Study end point

The primary outcome of interest was long-term, all-cause mortality. Information about post-discharge mortality tracking was available for all patients and was obtained by linking the institutional database with the National General Register Office.

The secondary endpoints were death during index hospitalization, incidence of new cerebrovascular accidents (CVA), re-exploration for bleeding, deep sternal wound infection and length of stay. CVA were defined as transient ischaemic attack or the occurrence of permanent stroke, diagnosed clinically and radiologically during the index hospitalization.

As sensitivity analysis, primary and secondary outcomes were investigated in the last decade (from 2009) in order to better understand the outcomes on the most recent cohort of SAVR patients.

Pre-specified subgroup analyses for the primary endpoint were age (<75 vs ≥75), gender and left ventricular ejection fraction.

### Statistical analysis

Shapiro–Wilk test was used to assess whether variables were well-modelled by a normal distribution. Centrality and dispersion for continuous variables were measured with mean ± SD or median and IQR. Categorical variables were described as frequency (%). Per the pre-specified statistical plan, differences in baseline characteristics between trainees and consultant group were evaluated with *t*-test for normally distributed variables or Wilcoxon rank-sum tests for non-normally distributed variables, and Pearson’s χ^2^ test for categorical variables.

To account for measured potential confounders, a propensity score (PS) based on a non-parsimonious logistic regression model was calculated for each patient. The covariates included in the model were age, gender, New York Heart Association (NYHA) functional class 3 or 4, Canadian Cardiovascular Society (CCS) class 3 or 4, diabetes mellitus, arterial hypertension, smoking, previous myocardial infarction, previous percutaneous coronary intervention, chronic kidney disease, chronic obstructive pulmonary disease, previous CVA, peripheral artery disease, preoperative atrial fibrillation, left ventricular ejection function (<50% or ≥50%), cardiogenic shock, body mass index, responsible consultant, the year of surgery and the priority of the procedure (elective versus urgent). The binary dependent variable was procedure performed by a trainee or a consultant. The treatment effect was analysed using propensity score matching (PSM). Pairs of patients were derived using 1:1 matching, with a calliper of width of 0.2 SDs of the logit of the PS by nearest-neighbour method. Standardized mean differences (SMD) were used to assess the balance of covariates between the 2 groups. A value higher than 0.10 was considered to indicate the presence of residual imbalance among variables. The quality of the match was also assessed graphically through a Love plot of SMD that assesses the balance of the variables between the 2 groups, and a mirror plot, that shows the ‘common support area’ for the spectrum of PS values between the 2 groups (Supplementary Material, Fig. S1).

Multivariable Cox regression was used to investigate the effect of trainee versus consultants on survival. This model was adjusted for all the variables already included in the PS model (‘doubly-robust’). The effect of first operator (trainee versus consultant) on long-term mortality was also investigated according to the stage of training of the trainee (early careers: years 1 and 2; mid-career: years 3 and 4; late career: years 5+) and according to 3 risk categories based on EuroSCORE [7]: low risk 0–2, medium risk 3–5 and high risk ≥6.

A generalized, linear model was used for short-term outcomes. This model was adjusted only for the EuroSCORE as the number of events of the short-term outcomes did not allow to force in the model all the variables used in the PS model. Therefore, we decided to adjust for the EuroSCORE as it is a comprehensive, risk-stratifying clinical variable.

To account for paired membership of patients included in the sample, cluster-robust standard errors were computed in the regression models. Paired *t*-test and Wilcoxon sign rank test were used to compare outcomes after PSM to account for the dependency of pairs.

To investigate the potential presence of calendar time bias, we also stratified the analysis according to 3 eras: 1996–2001, 2002–2009 and 2010–2017.

In all the analyses, the consultant group was used as the reference. There was no pre-specified plan to adjust for multiple comparisons. Significance testing was not performed for subgroup analyses. For these analyses, only estimates of the association between first operator and outcomes and corresponding 95% confidence intervals are shown and the results are exploratory. All *P*-values are 2-sided and *P*-values <0.05 were considered to indicate statistical significance. Statistical analysis was performed using R version 4.0.0 (packages: tableone, MatchIt, lmtest, ggplot2, survminer and sjplot).

## RESULTS

### Study population

From 28 761 patients included in the original dataset, we identified 3090 patients for the final analysis who underwent isolated SAVR during the study period (Supplementary Material, Fig. S2). Of those, 2220 (71.8%) were operated on by a consultant and 870 (28%) by a trainee. There was a total of 25 consultants and the median number of SAVR performed by them was 52 (19–133). The stage of surgical training was reported in 542 (62.3%) cases and there were 29 (5%) trainees in the first 2 years of training (early career), 145 (27%) in the 3rd and 4th year (mid-career) and 368 (68%) in the last years (late career). One hundred and nine procedures were performed by unsupervised trainees. Of those, the training stage was reported in 89 and most of them (85%) were senior trainees. The median number of SAVR performed by trainees was 4 (1–18).

The proportion of procedures performed by trainees showed a downwards trend from 41.5% of cases in the first era to 25.9% in the last era (Supplementary Material, Fig. S3). No significant changes were found in the proportions of trainees in each training stage performing SAVR, as most of the procedures were performed by late-career trainees throughout the years (Supplementary Material, Fig. S4).

Preoperative patient demographics and comorbidities before and after PSM are presented in Table 1. Patients operated on by consultants, when compared to patients operated on by trainees had a higher rate of female gender (45.1% vs 36.8%), NYHA class 3 or 4 (46.3% vs 40.3%), chronic kidney disease (2.5% vs 1.0%), chronic obstructive pulmonary disease (16.4% vs 12.1%), previous CVA (9.4% vs 7.0%) and left ventricular ejection function  < 50% (20% vs 15.7%). After PSM, differences in baseline characteristics were comparable between the 2 groups (SMD < 0.10; Table 1; Supplementary Material, Fig. S5).

**Table 1:** Patients baseline characteristics before and after propensity score matching

	Unmatched sample				Matched sample			
	Consultant	Trainee	*P* ^a^	SMD	Consultant	Trainee	*P* ^a^	SMD
*N*	2220	870			870	870		
Age, years, mean (SD)	68.76 (11.90)	68.44 (11.00)	0.50	0.028	68.10 (12.22)	68.44 (11.00)	0.54	0.029
Female, *n* (%)	1001 (45.1)	320 (36.8)	<0.001	0.170	314 (36.1)	320 (36.8)	0.80	0.014
NYHA class 3 or 4, *n* (%)	1028 (46.3)	351 (40.3)	0.003	0.121	348 (40.0)	351 (40.3)	0.92	0.007
CCS class 3 or 4, *n* (%)	244 (11.0)	89 (10.2)	0.58	0.025	96 (11.0)	89 (10.2)	0.64	0.026
MI, *n* (%)	110 (5.0)	29 (3.3)	0.06	0.081	33 (3.8)	29 (3.3)	0.70	0.025
PCI, *n* (%)	63 (2.8)	23 (2.6)	0.86	0.012	24 (2.8)	23 (2.6)	1.00	0.007
Diabetes, *n* (%)	309 (13.9)	109 (12.5)	0.34	0.041	106 (12.2)	109 (12.5)	0.88	0.010
Hypertension, *n* (%)	1229 (55.4)	488 (56.1)	0.74	0.015	482 (55.4)	488 (56.1)	0.81	0.014
Smoking (%)			0.049	0.100			0.32	0.072
Never smoked	1078 (48.6)	407 (46.8)			418 (48.0)	407 (46.8)		
Former smoker	958 (43.2)	409 (47.0)			385 (44.3)	409 (47.0)		
Active smoker	184 (8.3)	54 (6.2)			67 (7.7)	54 (6.2)		
CKD, *n* (%)	55 (2.5)	9 (1.0)	0.017	0.110	9 (1.0)	9 (1.0)	1.00	<0.001
COPD, *n* (%)	364 (16.4)	105 (12.1)	0.003	0.124	118 (13.6)	105 (12.1)	0.39	0.045
Stroke, *n* (%)	208 (9.4)	61 (7.0)	0.043	0.086	60 (6.9)	61 (7.0)	1.00	0.005
PVD, *n* (%)	82 (3.7)	38 (4.4)	0.44	0.034	50 (5.7)	38 (4.4)	0.23	0.063
Preoperative AF, *n* (%)	215 (9.7)	82 (9.4)	0.88	0.009	70 (8.0)	82 (9.4)	0.35	0.049
LVEF <50%, *n* (%)	445 (20.0)	137 (15.7)	0.007	0.112	129 (14.8)	137 (15.7)	0.64	0.026
BMI, mean (SD)	27.42 (5.29)	26.84 (5.31)	0.007	0.108	26.95 (5.12)	26.84 (5.31)	0.66	0.021
Preoperative shock, *n* (%)	6 (0.3)	0 (0.0)	0.28	0.074	0 (0.0)	0 (0.0)	NA	<0.001
Urgent, *n* (%)	621 (28.0)	151 (17.4)	<0.001	0.256	163 (18.7)	151 (17.4)	0.49	0.036
Euroscore, mean (SD)	5.65 (2.40)	5.29 (2.14)	<0.001	0.159	5.29 (2.39)	5.29 (2.14)	0.98	0.002

a *T*-test student for continuous variables; Chi-square test for categorical variables.

AF: atrial fibrillation; BMI: body mass index; CCS: Canadian Cardiovascular Society; CKD: chronic kidney disease; COPD: chronic obstructive pulmonary disease; LVEF: left ventricular ejection fraction; MI: myocardial infarction; NYHA: New York Heart Association; PCI: percutaneous coronary intervention; PVD: peripheral vascular disease.

Across the eras, the risk profile of patients undergoing SAVR increased and in both groups as patients were progressively older and with a major burden of comorbidities, such as diabetes, hypertension and obesity (Supplementary Material Table S1).

### Intraoperative data

Intraoperative data in the 2 groups after PSM are reported in Supplementary Material, Table S2. There were no differences regarding aortic valve haemodynamic, the type of implanted aortic valve and the ring size of the implanted prostheses. Patients operated on by consultant were more likely to present with active aortic valve endocarditis and to undergo shorter cardiopulmonary bypass and cross-clamp times when compared to patients operated on by trainees.

### Short-term outcomes

The operative and perioperative outcomes are presented in Table 2. There were no differences in short-term outcomes between patients operated on by consultants versus trainees. Trainees as the first operator did not increase the risk of short-term outcomes (Table 2; Supplementary Material, Tables S3–S7). These findings were also confirmed for cases where unsupervised trainees were the first operator (Supplementary Material, Table S8).

**Table 2.** Short-term outcomes in the overall population and in the 2 groups after propensity score matching

	Propensity score-matched sample			Adjusted estimates		
	Consultants	Trainees	*P* ^a^	Estimate	95% CI	*P*
*N*	870	870				
Return to theatre for bleeding, *n* (%)	33 (3.8)	35 (4.0)	0.90	OR: 1.07	0.66–1.74	0.79
DSWI, *n* (%)	2 (0.2)	1 (0.1)	1.00	OR: 0.52	0.02–5.44	0.59
CVA, *n* (%)			0.89	OR: 1.03	0.40–2.66	0.95
Transient stroke	4 (0.5)	3 (0.3)				
Permanent stroke	5 (0.6)	6 (0.7)				
In-hospital death, *n* (%)	10 (1.1)	10 (1.1)	1.00	OR: 1.04	0.42–2.57	0.92
LOS, mean (SD)	9.46 (7.07)	9.14 (6.36)	0.33	MD: −0.31	−0.94–0.31	0.33

a Wilcoxon signed-rank paired test; paired *t*-test.

CI: confidence interval; CVA: cerebrovascular accidents; DSWI: deep sternal wound infection; LOS: length of stay; MD: mean difference; OR: odds ratio.

In the overall population, there were 43 (1.4%) deaths during the index hospitalization, of whom 33 were in the consultant group and 10 in the trainee group. Among these, one occurred in an early career trainee, 2 in mid-career trainees and 3 in late-career trainees. No information about training stage was available for the remaining 4 deaths.

No differences in the short-term outcomes were also found when the analysis was limited to urgent SAVR (Supplementary Material, S9).

There were no differences in discharge destinations, with most patients in both groups being discharged home and <3% to other acute hospitals.

Finally, the event rate of the short-term outcomes was comparable between consultant and trainee cases throughout the eras (Supplementary Material, Table S10).

### Long-term mortality

**Figure 1:**
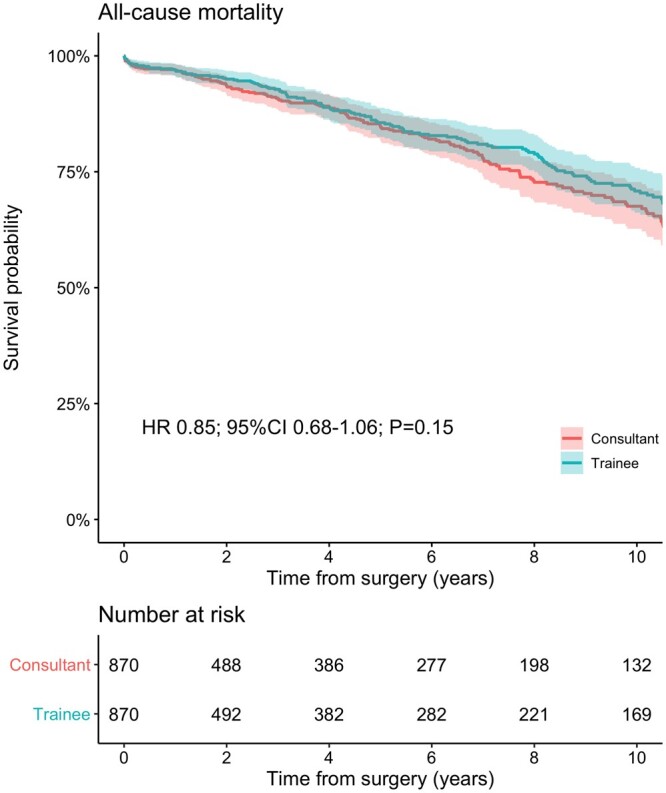
Kaplan–Meier curves describing the cumulative survival probability in patients undergoing isolated surgical aortic valve replacement performed by consultants or trainees, after propensity score matching.

**Figure 2:**
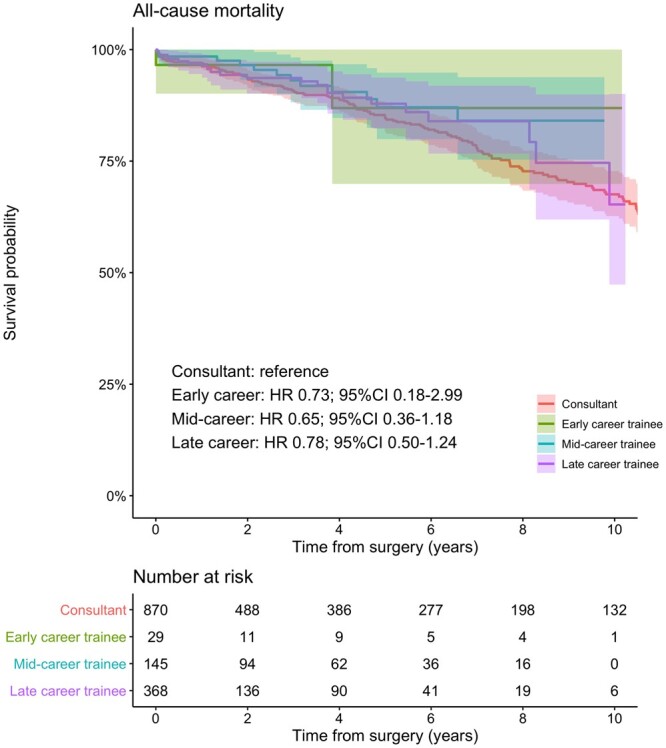
Kaplan–Meier curves describing the cumulative survival probability in patients undergoing isolated surgical aortic valve replacement performed by consultants or trainees stratified by trainees’ career stage, after propensity score matching.

The mean follow-up time in the overall population was 4.1 (±4.5) years, 4.6 (±4.9) in the trainee group and 3.9 (±4.4) in the consultant group. The survival probability at 1, 5 and 10 years was 96.9% (95.6%–98.2%) vs 96.9% (95.5%–98.2%), 84.8% (81.7%–88%) vs 85.6% (82.5%–88.8%) and 67.6% (62.7%–72.8%) vs 70.8% (66.2%–75.8%) in the consultant and trainee group, respectively (Fig. 1). There was a comparable risk of late death between cases performed by trainees versus consultant (HR 0.85; 95% CI 0.68–1.06; *P* = 0.15; Supplementary Material, Table S11). Moreover, throughout all training stages, trainee cases were not associated with a higher risk of long-term mortality (early career: HR 0.73; 95% CI 0.18–2.99; mid-career: HR 0.65; 95% CI 0.36–1.18; late career: HR 0.78; 95% CI 0.50–1.24; Fig. 2). Similarly, the survival outcome of unsupervised trainees was not associated with a higher risk of mortality compared to the consultant group (HR 0.81; 95% CI 0.45–1.43; Supplementary Material, Fig. S6).

**Figure 3:**
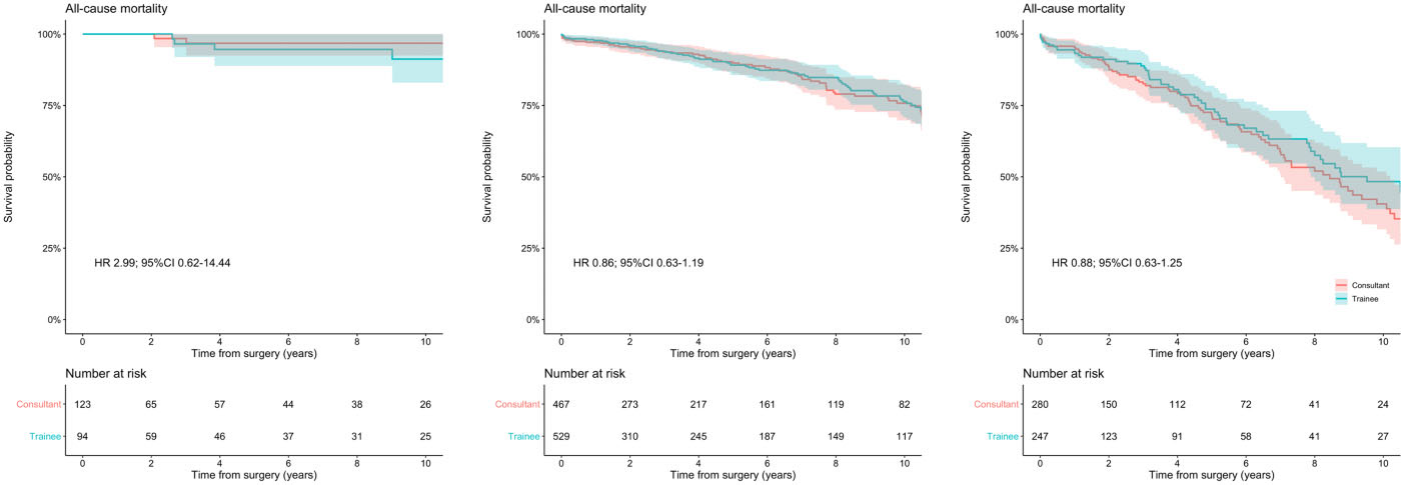
Kaplan–Meier curves describing the cumulative survival probability in patients undergoing isolated surgical aortic valve replacement performed by consultants or trainees in the low-risk (left), mid-risk (central) and high-risk (right) cohorts, after propensity score matching.

Moreover, there was no difference in the risk of late death was shown between trainees and consultant cases when the analysis was stratified according to the predicted surgical risk based on the EuroSCORE: low-risk cases (HR 2.99; 95% CI 0.62–14.44; Fig. 3 left), intermediate-risk cases (HR 0.86; 95% CI 0.63–1.19; Fig. 3 middle) and high-risk cases (HR 0.88; 95% CI 0.63–1.25; Fig. 3 right). Also, no differences were found in the risk of late death between consultants and trainees when the analysis was limited to urgent SAVR cases (HR 0.67; 0.38–1.16; Supplementary Material, Fig. S7).

The risk of long-term mortality did not change throughout the eras: HR 0.98; 95% CI 0.71–1.36 in 1996–2001; HR 0.78; 95% CI 0.55–1.11 in 2002–2009; and HR 0.91; 95% CI 0.30–2.79 in 2010–2017.

**Figure 4:**
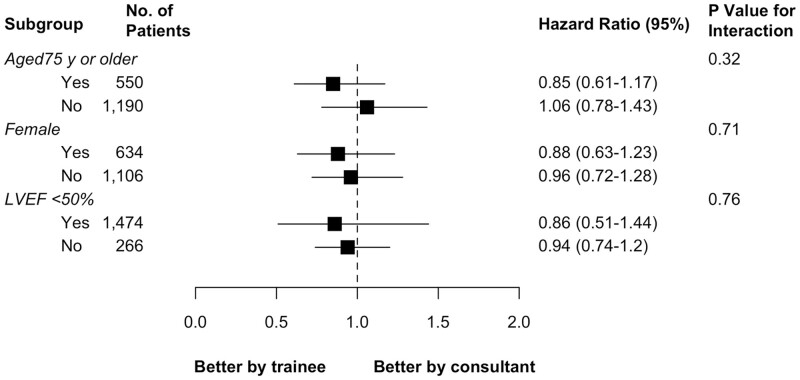
Effect modifiers of the association between first operator status (trainee versus consultant) and long-term mortality. LVEF: left ventricular ejection fraction.

Finally, the risk of late death associated with the first operator being a trainee versus a consultant was not different across the subgroups and no interaction was found between first operator status and age, gender and reduced left ventricular ejection function (Fig. 4).

## DISCUSSION

In this study, we demonstrated that short-term clinical outcomes and long-term survival after isolated SAVR are not negatively affected by trainees acting as the first operator when compared with consultants. After adjusting for baseline risk factors, no statistically significant differences were found in in-hospital outcomes (death, new CVA, deep sternal wound infection, return to theatre for bleeding, length of stay) and late, all-cause mortality between the 2 groups. Moreover, no excess of late mortality was noted when the analysis was stratified across trainees’ career stage and when patients were split into 3 surgical risk groups according to the EuroSCORE. Also, unsupervised trainees without a consultant scrubbed in the operation lead to similar outcomes compared to supervised trainees and consultant cases. Stratifying the analysis according to 3 different eras to account for temporal variation in surgical techniques and patient care, we found no differences in terms of outcomes despite of an increase in the risk profile of patients.

To the best of our knowledge, this study is the one with the largest SAVR cases performed by trainees reported in the literature.

In a recent meta-analysis [8] of 6 studies (6236 patients) reporting the outcomes after SAVR performed by trainees versus consultants, the authors found similar perioperative mortality (OR 0.67; 95% CI 0.37–1.24) and no differences in terms of perioperative stroke, reoperation for bleeding and wound infection between the 2 groups. No pooled mid-term mortality was described as only one of the studies included reported it. The time in which studies were conducted ranged from 1977 up until 2013, with most of them before 2010. In our study, we included patients undergoing isolated SAVR from 1996 to 2017 and this allowed us to better characterize the changes in patients’ risk profile that has taken place recently and therefore, the impact of surgical training on this new high-risk, surgically complex cohort. As previously reported [1–3], in the latest years, patients undergoing SAVR were more likely to be older and present with more comorbidities. However, this increased risk profile did not impact on trainees’ outcomes comparable to the ones achieved by consultants.

More recently Szczechowicz *et al.* [9] reported on the short-term outcomes of 3077 patients. Of those, 118 patients underwent isolated SAVR performed by trainees. After PSM, the 30-day mortality and the incidence of postoperative complications were not significantly different between the 2 groups. Similarly, in the study by Luthra *et al.* [10], the perioperative outcomes of 639 patients operated on by trainees were comparable with the results achieved by consultants. It was not possible to find any study supporting the evidence that trainees acting as first operators were associated with worse outcomes. This may be related to the presence of publication bias and the reluctance towards publishing negative results.

Moreover, only 2 studies reported on the comparison of mid- and long-term mortality between trainees’ and consultants’ cases and no difference was found [11, 12]. Compared to these studies, we reported on longer mean follow-up outcome and demonstrated that the equipoise between trainees’ and consultants’ cases persists during a longer follow-up.

Our primary endpoint was late, all-cause mortality which is considered the most unbiased and strongest index of death in cardiovascular research. Indeed, in contrast to all-cause mortality, cause-specific mortality needs adjudication, and this may be difficult due to the presence of concomitant comorbidities, low autopsy rate and inadequate understanding of complex disease process [13].

This analysis supports the training of trainees as the first operator in SAVR despite of the increased high risk of patient population and complexity of procedures in recent years. Although we found longer cardiopulmonary bypass and cross-clamp times in the trainee group, this did not translate into the worst outcomes, suggesting that operative educational opportunities can be safely pursued.

There was an overall reduction in the proportion of cases performed by trainees from the first era to the last one. This reduction could be either the result of a greater reluctance of consultants to let trainees perform the cases, given the higher risk profile of patients, or the effect of the progressive advancement and adoption of interventional procedures, such as transcatheter aortic valve replacement, which can reduce the pool of available patients undergoing SAVR and therefore impact the overall exposure of trainees to SAVR. Our findings are especially important in the current era of progressive use of transcatheter aortic valve interventions. Given the safety of SAVR performed by trainees, surgical training programmes should strongly aim to keep securing a proper training in SAVR for the future generations of cardiac surgeons.

Our results were possible due to a structured, skill-oriented training programme during which trainees are progressively exposed to the surgical steps of each procedure until they can put all the pieces together and, assisted by a consultant, perform the whole procedure.

### Limitations

This study has some limitations. The first limitation is inherent to its nonrandomized and retrospective nature. Although we tried to account for difference among the 2 groups through the application of PSM, this method is only able to balance measured confounders and not unmeasured confounders, which are more difficult to quantify and are mainly based on the ‘eyeball’ test (e.g. patient frailty or inactivity). Therefore, there may persist a certain degree of selection bias and potential confounding which could have influenced our findings. Secondly, there were no data regarding the ‘cross-over’ from trainee to consultant designation as the first operator. This shift could have happened in cases presenting unexpected findings or intraoperative complications and could have led to an overestimation of trainee performance. However, we believe that this event did not occur to a significant extent. Thirdly, we do not have data regarding the rate of pacemaker implantation, postoperative blood transfusion and prosthetic valve performance during the follow-up period. Finally, we do not have data regarding the factors which helped the consultant to decide whether to let the trainee perform the procedure. There are certain settings, such as patients with deep chest, endocarditis or mediastinal adhesions which may prevent the trainees from performing the surgery. However, this decision relies strongly on the expertise of both the responsible surgeon and the trainee and therefore no absolute characteristics that preclude the trainees from performing the surgery can be discussed.

## CONCLUSION

In conclusion, isolated SAVR is a safe and reproducible technique, and its outcomes are not significantly different when trainees acted as the first operator, regardless of their training stage and patients risk profile.

## SUPPLEMENTARY MATERIAL

Supplementary material is available at *EJCTS* online.

## Funding

This study was supported by the British Heart Foundation and NIHR Biomedical Research Centre at University Hospitals Bristol and Weston NHS Foundation Trust and the University of Bristol.

**Conflict of interest:** none declared.

## Author contributions

**Arnaldo Dimagli:** Conceptualization; Data curation; Formal analysis; Methodology; Resources; Supervision; Writing—original draft; Writing—review & editing. **Shubhra Sinha:** Conceptualization; Methodology; Writing—original draft; Writing—review & editing. **Umberto Benedetto:** Formal analysis; Methodology; Supervision; Writing—original draft; Writing—review & editing. **Massimo Caputo:** Supervision; Writing—original draft; Writing—review & editing. **Gianni D. Angelini:** Funding acquisition; Resources; Supervision; Writing—original draft; Writing—review & editing.

## Reviewer information

European Journal of Cardio-Thoracic Surgery thanks Andrew E. Newcomb, Gopal K. R. Soppa and the other anonymous reviewer(s) for their contribution to the peer review process of this article.

## Supplementary Material

ezab328_Supplementary_Data

## Notes

Presented at the 34th Annual Meeting of the European Association for Cardio-Thoracic Surgery, Barcelona, Spain, 8–10 October 2020.

## ABBREVIATIONS

CVA: Cerebrovascular accidents
PS: Propensity score
PSM: Propensity score matching
SAVR: Surgical aortic valve replacement
SMD: Standardized mean differences
