# Supplementary material for: The impact of surgical training on early and long-term outcomes after isolated aortic valve surgery
Source: Eur J Cardiothorac Surg. 2021 Aug 6;61(1):180–6. doi: 10.1093/ejcts/ezab328 (PMC8715849; doi:10.1093/ejcts/ezab328)
Supplement: ezab328_Supplementary_Data [file ezab328_supplementary_data.docx]

**Supplementary material**

**The impact of surgical training on early and long-term outcomes after isolated aortic valve surgery.**

Arnaldo Dimagli et al.

**Contents**

**Supplementary Tables**

**Supplementary Table 1.** Baseline characteristics of patients included in the study in each era stratified by first operator.

**Supplementary Table 2.** Intraoperative data in the overall population and in the two groups after propensity score matching.

**Supplementary Table 3.** Results of the logistic regression model for return to theatre for bleeding

**Supplementary Table 4.** Results of the logistic regression model for deep sternal wound infection.

**Supplementary Table 5.** Results of the logistic regression model for in-hospital cerebrovascular accidents.

**Supplementary Table 6.** Results of the logistic regression model for in-hospital death.

**Supplementary Table 7.** Results of the linear regression model for in-hospital length of stay.

**Supplementary Table 8.** Short-term outcomes compared between supervised and unsupervised trainees and consultants.

**Supplementary Table 9.** Short-term outcomes between urgent patients operated on by consultants vs trainees.

**Supplementary Table 10.** Short-term outcomes in the consultant and trainee groups stratified by eras.

**Supplementary Table 11.** Result of the Cox regression model for long-term mortality.

**Supplementary Figures**

**Supplementary Figure 1.** Mirror plot showing the distribution of the propensity scores before and after matching and highlighting the “common support area”.

**Supplementary Figure 2.** Flowchart of the patients included in the study.

**Supplementary Figure 3.** Proportion of cases performed by trainees as first operator out of the total number of surgical aortic valve replacement performed from 1996-2017.

**Supplementary Figure 4.** Proportion of surgical aortic valve procedures performed by trainees in each of the three career stage: early (1^st^-2^nd^ year of training), mid (3^rd^-4^th^ year of training) and late (after 5^th^ year of training).

**Supplementary Figure 5.**  Love plot showing the changes in standardized mean difference before and after propensity score matching. It demonstrates that the balance of covariates was improved on all variables, which are below the threshold of 0.1 of absolute mean difference.

**Supplementary Figure 6.** Kaplan-Meier curves describing the cumulative survival probability in patients undergoing isolated surgical aortic valve replacement performed by consultants or unsupervised trainees.

**Supplementary Figure 7.** Kaplan-Meier curves describing the cumulative survival probability in patients undergoing urgent isolated surgical aortic valve replacement performed by consultants or trainee.

**Supplementary Table 1.** Baseline characteristics of patients included in the study in each era stratified by first operator.

|  | | 1996-2001 | | | 2002-2009 | | | 2010-2017 | | |
| --- | --- | --- | --- | --- | --- | --- | --- | --- | --- | --- |
|  | | Consultant | Trainee | P^1^ | Consultant | Trainee | P^1^ | Consultant | Trainee | P^1^ |
| n | | 142 | 195 |  | 324 | 269 |  | 404 | 406 |  |
| AGE, years, mean (SD) | | 64.45 (12.31) | 66.72 (10.02) | 0.062 | 68.17 (12.21) | 67.44 (11.79) | 0.461 | 69.34 (11.98) | 69.94 (10.74) | 0.453 |
| FEMALE, n (%) | | 62 ( 43.7) | 83 ( 42.6) | 0.929 | 117 ( 36.1) | 92 ( 34.2) | 0.69 | 135 ( 33.4) | 145 ( 35.7) | 0.539 |
| NYHA class 3 or 4, n (%) | | 61 ( 43.0) | 87 ( 44.6) | 0.848 | 127 ( 39.2) | 101 ( 37.5) | 0.744 | 160 ( 39.6) | 163 ( 40.1) | 0.931 |
| CCS class 3 or 4, n (%) | | 28 ( 19.7) | 29 ( 14.9) | 0.305 | 37 ( 11.4) | 27 ( 10.0) | 0.684 | 31 ( 7.7) | 33 ( 8.1) | 0.913 |
| MI, n (%) | | 2 ( 1.4) | 3 ( 1.5) | 1 | 13 ( 4.0) | 8 ( 3.0) | 0.647 | 18 ( 4.5) | 18 ( 4.4) | 1 |
| PCI, n (%) | | 142 (100.0) | 195 (100.0) | NA | 6 ( 1.9) | 6 ( 2.2) | 0.974 | 18 ( 4.5) | 17 ( 4.2) | 0.988 |
| DIABETES, n (%) | | 5 ( 3.5) | 17 ( 8.7) | 0.092 | 39 ( 12.0) | 27 ( 10.0) | 0.522 | 62 ( 15.3) | 65 ( 16.0) | 0.871 |
| HYPERTENSION, n (%) | | 69 ( 48.6) | 91 ( 46.7) | 0.811 | 185 ( 57.1) | 147 ( 54.6) | 0.606 | 228 ( 56.4) | 250 ( 61.6) | 0.157 |
| SMOKING (%) |  | 0.069 | | | 0.954 | | | 0.344 | | |
| Never smoked | | 80 ( 56.3) | 95 ( 48.7) |  | 134 ( 41.4) | 110 ( 40.9) |  | 204 ( 50.5) | 202 ( 49.8) |  |
| Former smoker | | 46 ( 32.4) | 86 ( 44.1) |  | 168 ( 51.9) | 139 ( 51.7) |  | 171 ( 42.3) | 184 ( 45.3) |  |
| Active smoker | | 16 ( 11.3) | 14 ( 7.2) |  | 22 ( 6.8) | 20 ( 7.4) |  | 29 ( 7.2) | 20 ( 4.9) |  |
| CKD, n (%) | | 3 ( 2.1) | 2 ( 1.0) | 0.72 | 2 ( 0.6) | 3 ( 1.1) | 0.834 | 4 ( 1.0) | 4 ( 1.0) | 1 |
| COPD, n (%) | | 13 ( 9.2) | 17 ( 8.7) | 1 | 41 ( 12.7) | 28 ( 10.4) | 0.471 | 64 ( 15.8) | 60 ( 14.8) | 0.747 |
| STROKE, n (%) | | 9 ( 6.3) | 10 ( 5.1) | 0.813 | 25 ( 7.7) | 22 ( 8.2) | 0.956 | 26 ( 6.4) | 29 ( 7.1) | 0.795 |
| PVD, n (%) | | 4 ( 2.8) | 7 ( 3.6) | 0.933 | 13 ( 4.0) | 11 ( 4.1) | 1 | 33 ( 8.2) | 20 ( 4.9) | 0.085 |
| Preoperative AF, n (%) | | 8 ( 5.6) | 19 ( 9.7) | 0.242 | 25 ( 7.7) | 24 ( 8.9) | 0.703 | 37 ( 9.2) | 39 ( 9.6) | 0.922 |
| LVEF <50%, n (%) | | 26 ( 18.3) | 32 ( 16.4) | 0.757 | 41 ( 12.7) | 39 ( 14.5) | 0.594 | 62 ( 15.3) | 66 ( 16.3) | 0.796 |
| BMI, mean (SD) | | 23.57 (6.10) | 23.99 (6.06) | 0.524 | 27.64 (4.69) | 27.30 (5.26) | 0.405 | 27.60 (4.59) | 27.91 (4.41) | 0.317 |
| Preoperative shock, n (%) | | 0 (0.0) | 0 (0.0) | NA | 0 (0.0) | 0 (0.0) | NA | 0 (0.0) | 0 (0.0) | NA |
| Urgent, n (%) | | 32 ( 22.5) | 34 ( 17.4) | 0.305 | 64 ( 19.8) | 51 ( 19.0) | 0.889 | 67 ( 16.6) | 66 ( 16.3) | 0.975 |
| Euroscore, mean (SD) | | 5.06 (2.17) | 5.16 (1.95) | 0.633 | 5.42 (2.33) | 5.17 (2.12) | 0.185 | 5.28 (2.50) | 5.43 (2.23) | 0.363 |

^1^ T-test student for continuous variables; Chi-square test for categorical variables.

NYHA New York Heart Association; CCS Canadian Cardiovascular Society; MI myocardial infarction; PCI percutaneous coronary intervention; CKD chronic kidney disease; COPD chronic obstructive pulmonary disease; PVD peripheral vascular disease; AF atrial fibrillation; LVEF left ventricular ejection fraction; BMI body mass index.

**Supplementary Table 2.** Intraoperative data in the overall population and in the two groups after propensity score matching.

|  |  | Consultant | Trainee | P^1^ |
| --- | --- | --- | --- | --- |
| n |  | 870 | 870 |  |
| Valve hemodynamic, n (%) | Stenosis | 462 (73.9) | 519 (75.8) | 0.57 |
|  | Regurgitation | 86 (13.8) | 81 (11.8) |  |
|  | Mixed | 77 (12.3) | 85 (12.4) |  |
| Active endocarditis, n(%) |  | 17 ( 2.0) | 5 ( 0.6) | 0.018 |
| Valve implant type, n (%) | Mechanical | 244 (28.0) | 255 (29.3) | 0.60 |
|  | Biological | 626 (72.0) | 615 (70.7) |  |
| Implanted ring size, n (%) | 17 | 2 ( 0.2) | 2 ( 0.2) | 0.05 |
|  | 19 | 147 (17.0) | 111 (12.8) |  |
|  | 21 | 268 (30.9) | 300 (34.6) |  |
|  | 22 | 1 ( 0.1) | 0 ( 0.0) |  |
|  | 23 | 254 (29.3) | 291 (33.6) |  |
|  | 24 | 1 ( 0.1) | 0 ( 0.0) |  |
|  | 25 | 129 (14.9) | 115 (13.3) |  |
|  | 26 | 1 ( 0.1) | 0 ( 0.0) |  |
|  | 27 | 57 ( 6.6) | 35 ( 4.0) |  |
|  | 29 | 7 ( 0.8) | 11 ( 1.3) |  |
|  | 31 | 0 ( 0.0) | 1 ( 0.1) |  |
| Cardiopulmonary bypass time, min, median (IQR) |  | 82 (71-95) | 100 (89-113) | <0.001 |
| Cross-clamp time, min, median (IQR) |  | 60 (53-70) | 76 (67-86) | <0.001 |

^1^Wilcoxon signed rank paired test; Paired t-test. IQR, interquartile range.

**Supplementary Table 3.** Results of the logistic regression model for return to theatre for bleeding

|  | **Return to theatre for bleeding** | | |
| --- | --- | --- | --- |
| *Predictors* | *Odds Ratios* | *CI* | *p* |
| (Intercept) | 0.02 | 0.01 – 0.05 | **<0.001** |
| OPERATOR | 1.07 | 0.66 – 1.74 | 0.788 |
| Euroscore | 1.10 | 0.99 – 1.23 | 0.071 |
| Observations | 1740 | | |
| R^2^ Tjur | 0.002 | | |

**Supplementary Table 4.** Results of the logistic regression model for deep sternal wound infection.

|  | **Deep sternal wound infection** | | |
| --- | --- | --- | --- |
| *Predictors* | *Odds Ratios* | *CI* | *p* |
| (Intercept) | 0.00 | 0.00 – 0.01 | **<0.001** |
| OPERATOR | 0.52 | 0.02 – 5.44 | 0.592 |
| Euroscore | 1.28 | 0.79 – 2.02 | 0.303 |
| Observations | 1740 | | |
| R^2^ Tjur | 0.001 | | |

**Supplementary Table 5.** Results of the logistic regression model for in-hospital cerebrovascular accidents.

|  | **Cerebrovascular accidents** | | |
| --- | --- | --- | --- |
| *Predictors* | *Odds Ratios* | *CI* | *p* |
| (Intercept) | 0.00 | 0.00 – 0.01 | **<0.001** |
| OPERATOR | 1.03 | 0.40 – 2.66 | 0.950 |
| Euroscore | 1.26 | 1.03 – 1.53 | **0.024** |
| Observations | 1740 | | |
| R^2^ Tjur | 0.003 | | |

**Supplementary Table 6.** Results of the logistic regression model for in-hospital death.

|  | **In-hospital death** | | |
| --- | --- | --- | --- |
| *Predictors* | *Odds Ratios* | *CI* | *p* |
| (Intercept) | 0.00 | 0.00 – 0.01 | **<0.001** |
| OPERATOR | 1.04 | 0.42 – 2.57 | 0.924 |
| Euroscore | 1.32 | 1.09 – 1.59 | **0.004** |
| Observations | 1740 | | |
| R^2^ Tjur | 0.005 | | |

**Supplementary Table 7.** Results of the linear regression model for in-hospital length of stay.

|  | **Length of stay** | | |
| --- | --- | --- | --- |
| *Predictors* | *Estimates* | *CI* | *p* |
| (Intercept) | 6.42 | 5.57 – 7.27 | **<0.001** |
| OPERATOR | -0.31 | -0.94 – 0.31 | 0.325 |
| Euroscore | 0.57 | 0.44 – 0.71 | **<0.001** |
| Observations | 1722 | | |
| R^2^ / R^2^ adjusted | 0.038 / 0.037 | | |

**Supplementary Table 8.** Short-term outcomes compared between supervised and unsupervised trainees and consultants.

|  | Supervised trainees | Unsupervised trainess | Consultant | P^1^ |
| --- | --- | --- | --- | --- |
| n | 761 | 109 | 870 |  |
| Return to theatre for bleeding, n (%) | 31 ( 4.1) | 4 ( 3.7) | 33 ( 3.8) | 0.950 |
| DSWI, n (%) | 1 ( 0.1) | 0 ( 0.0) | 2 ( 0.2) | 0.807 |
| CVA, n (%) |  |  |  | 0.701 |
| Transient stroke | 2 ( 0.3) | 1 ( 0.9) | 4 ( 0.5) |  |
| Permanent stroke | 6 ( 0.8) | 0 ( 0.0) | 5 ( 0.6) |  |
| In-hospital death, n (%) | 8 ( 1.1) | 2 ( 1.8) | 10 ( 1.1) | 0.773 |
| LOS, mean (SD) | 9.07 (6.06) | 9.68 (8.18) | 9.46 (7.07) | 0.430 |

^1^ Wilcoxon signed rank paired test; Paired t-test.

OR odds ratio; CI confidence interval; DSWI, deep sternal wound infection; CVA, cerebrovascular accidents; LOS, length of stay; MD, mean difference

**Supplementary Table 9.** Short-term outcomes between urgent patients operated on by consultants vs trainees.

|  | Matched sample | | |
| --- | --- | --- | --- |
|  | Attending | Resident | P^1^ |
| n | 163 | 151 |  |
| Return to theatre for bleeding, n (%) | 9 ( 5.5) | 7 ( 4.6) | 0.92 |
| DSWI, n (%) | 0 (0.0) | 0 (0.0) | NA |
| CVA, n (%) |  |  | 0.65 |
| Transient stroke | 1 ( 0.6) | 1 ( 0.7) |  |
| Permanent stroke | 3 ( 1.8) | 1 ( 0.7) |  |
| In-hospital death, n (%) | 0 ( 0.0) | 5 ( 3.3) | 0.06 |
| LOS, mean (SD) | 10.32 (9.61) | 10.54 (7.82) | 0.83 |

^1^ Wilcoxon signed rank paired test; Paired t-test.

OR odds ratio; CI confidence interval; DSWI, deep sternal wound infection; CVA, cerebrovascular accidents; LOS, length of stay; MD, mean difference

**Supplementary Table 10.** Short-term outcomes in the consultant and trainee groups stratified by eras.

|  | 1996-2001 | | | 2002-2009 | | | 2010-2017 | | |
| --- | --- | --- | --- | --- | --- | --- | --- | --- | --- |
|  | Consultant | Trainee | P^1^ | Consultant | Trainee | P | Consultant | Trainee | P |
| n | 142 | 195 |  | 324 | 269 |  | 404 | 406 |  |
| Return to theatre for bleeding, n (%) | 9 ( 6.3) | 13 ( 6.7) | 1 | 12 ( 3.7) | 8 ( 3.0) | 0.794 | 12 (3.0) | 14 (3.4) | 0.852 |
| DSWI, n (%) | 0 (0.0) | 0 (0.0) | NA | 1 ( 0.3) | 1 ( 0.4) | 1 | 1 (0.2) | 0 (0.0) | 0.998 |
| CVA, n (%) |  |  | 0.229 |  |  | 0.676 |  |  | 0.246 |
| Transient stroke | 0 ( 0.0) | 1 ( 0.5) |  | 4 ( 1.2) | 2 ( 0.7) |  | 0 (0.0) | 0 (0.0) |  |
| Permanent stroke | 0 ( 0.0) | 3 ( 1.5) |  | 2 ( 0.6) | 3 ( 1.1) |  | 3 (0.7) | 0 (0.0) |  |
| In-hospital death, n (%) | 0 ( 0.0) | 3 ( 1.5) | 0.369 | 6 ( 1.9) | 4 ( 1.5) | 0.981 | 4 (1.0) | 3 (0.7) | 0.995 |
| LOS, mean (SD) | 9.80 (7.54) | 9.41 (5.16) | 0.566 | 9.84 (6.92) | 9.57 (7.57) | 0.645 | 9.02 (7.00) | 8.72 (5.96) | 0.525 |

^1^ Wilcoxon signed rank paired test; Paired t-test.

OR odds ratio; CI confidence interval; DSWI, deep sternal wound infection; CVA, cerebrovascular accidents; LOS, length of stay; MD, mean difference

**Supplementary Table 11.** Result of the Cox regression model for long-term mortality.

|  | **Long-term mortality** | | |
| --- | --- | --- | --- |
| *Predictors* | *Estimates* | *CI* | *p* |
| OPERATOR | 0.85 | 0.68 – 1.06 | 0.148 |
| AGE | 1.08 | 1.07 – 1.10 | **<0.001** |
| FEMALE | 0.89 | 0.70 – 1.14 | 0.329 |
| CCS34 | 0.86 | 0.62 – 1.20 | 0.363 |
| NYHA34 | 1.32 | 1.04 – 1.67 | **0.021** |
| MI | 1.78 | 0.97 – 3.28 | 0.058 |
| PCI | 0.28 | 0.04 – 2.05 | 0.198 |
| DIABETES | 1.23 | 0.84 – 1.79 | 0.328 |
| HYPERTENSION | 1.17 | 0.93 – 1.47 | 0.179 |
| SMOKING | 1.11 | 0.91 – 1.36 | 0.303 |
| CKD | 1.93 | 0.83 – 4.47 | 0.136 |
| COPD | 1.38 | 0.99 – 1.92 | 0.057 |
| STROKE | 1.36 | 0.93 – 1.99 | 0.091 |
| PVD | 1.17 | 0.71 – 1.93 | 0.523 |
| AF | 1.81 | 1.31 – 2.51 | **0.001** |
| LVEF | 1.13 | 0.84 – 1.53 | 0.428 |
| BMI | 0.98 | 0.96 – 1.00 | 0.072 |
| responsible_cons | 1.00 | 1.00 – 1.00 | 0.210 |
| PRIORITY | 0.91 | 0.69 – 1.22 | 0.519 |
| Observations | 1740 | | |
| R^2^ Nagelkerke | 0.516 | | |

CCS Canadian Cardiovascular scale; NYHA New York Heart Association; MI myocardial infarction; PCI percutaneous coronary intervention; CKD chronic kidney disease; COPD chronic obstructive pulmonary disease; PVD peripheral vascular disease; AF atrial fibrillation; LVEF left ventricular ejection fraction.

**Supplementary Figure 1.** Mirror plot showing the distribution of the propensity scores before and after matching and highlighting the “common support area”.


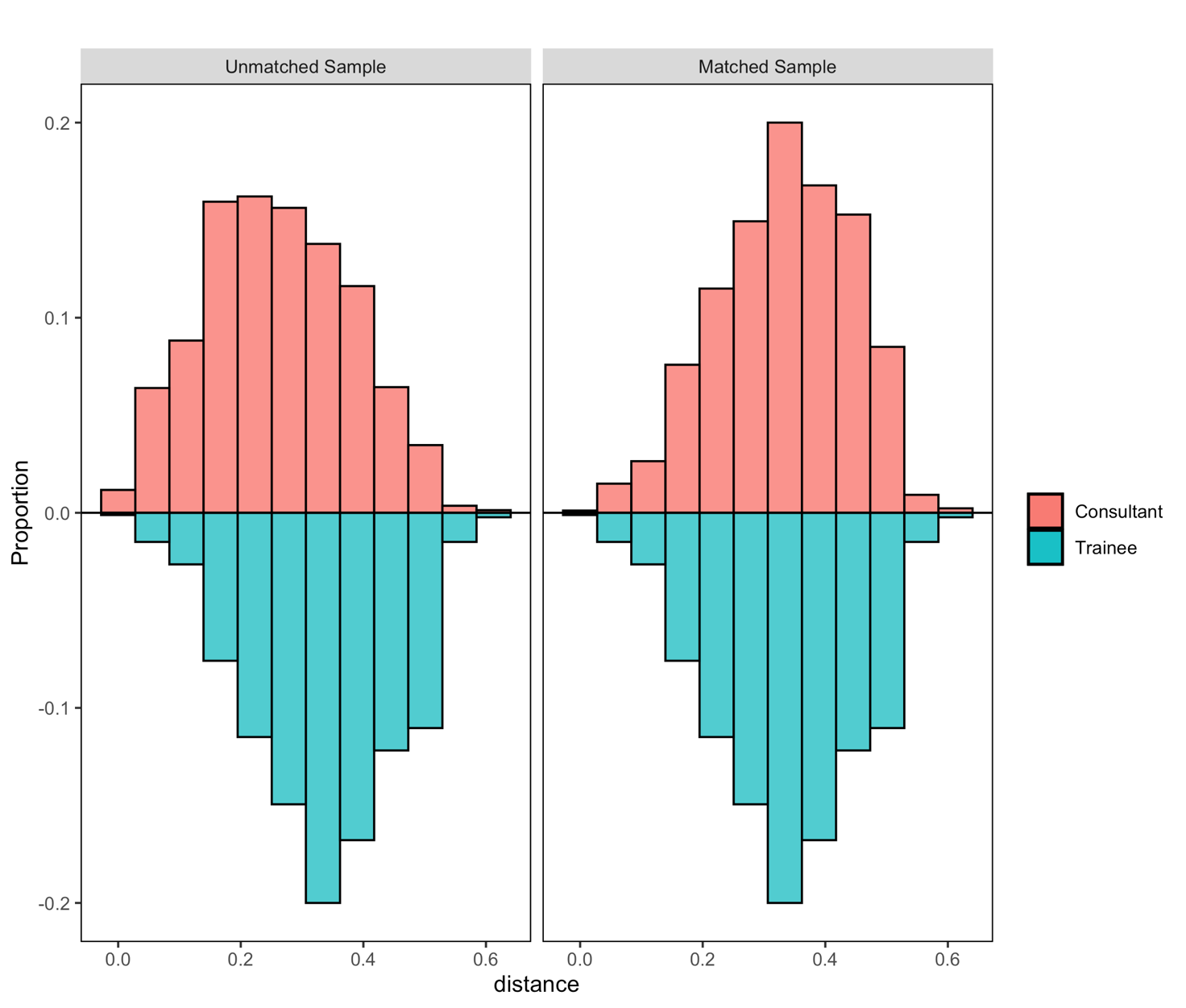


**Supplementary Figure 2.** Flowchart of the patients included in the study.


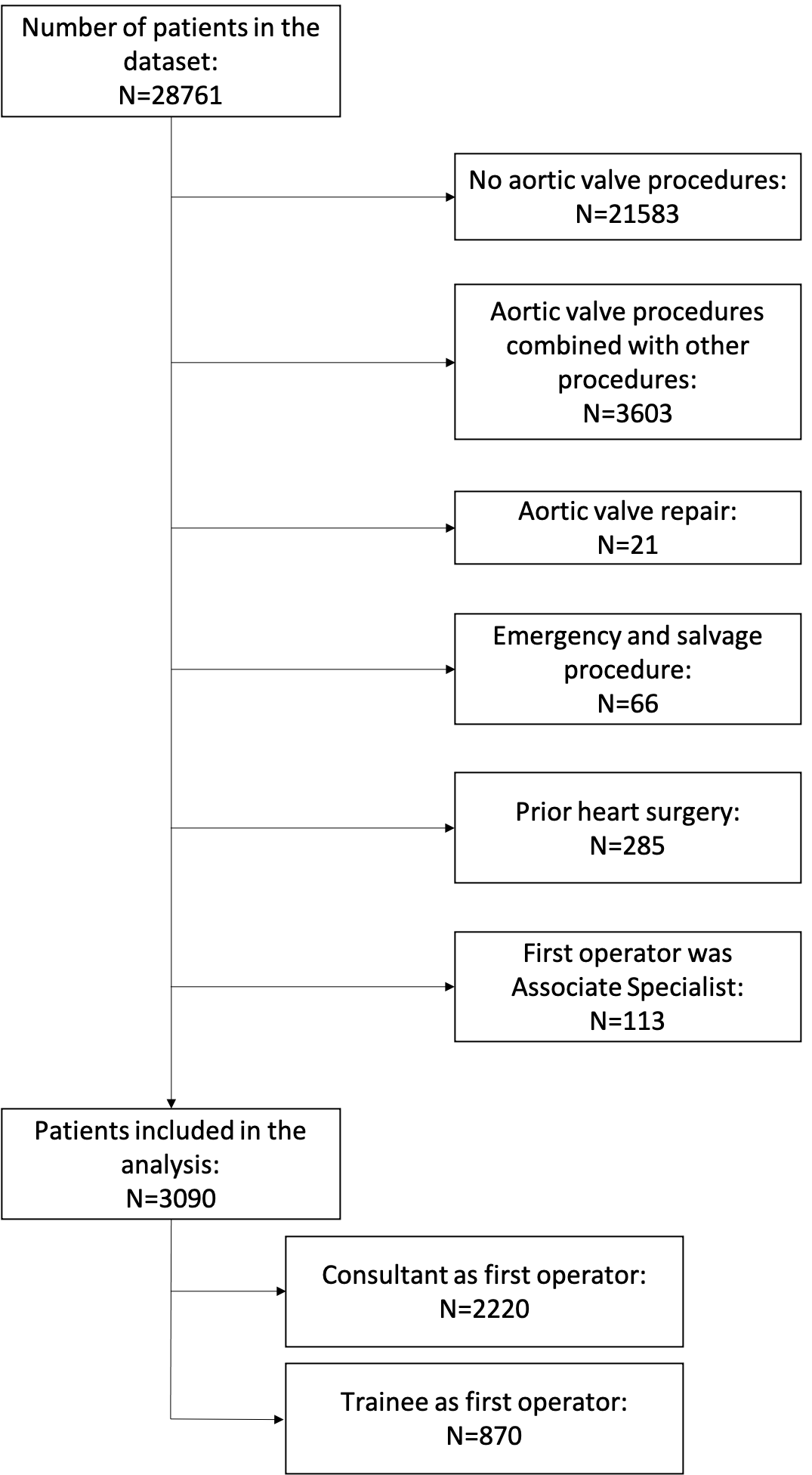


**Supplementary Figure 3.** Proportion of cases performed by trainees as first operator out of the total number of surgical aortic valve replacement performed from 1996-2017.


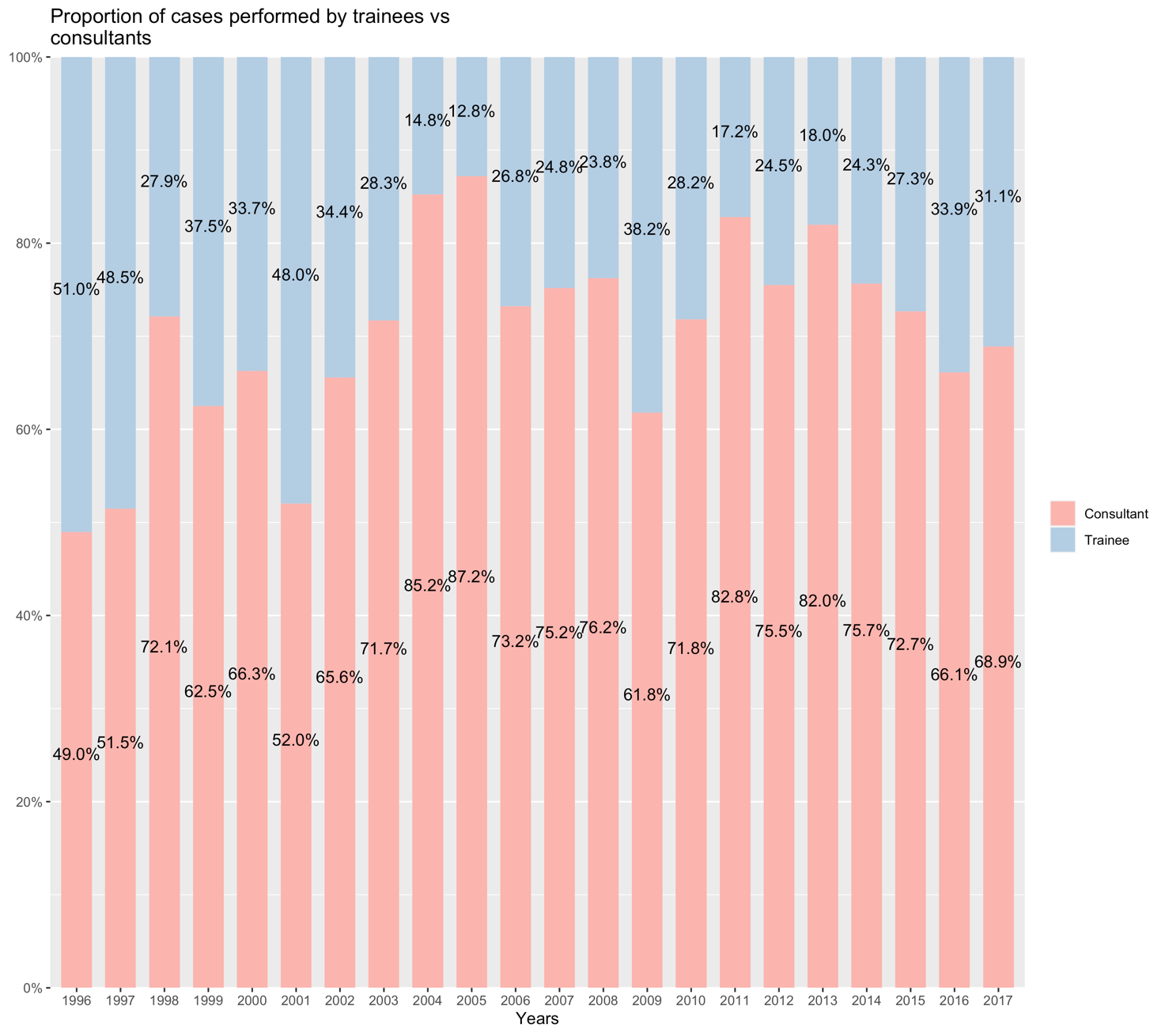


**Supplementary Figure 4.** Proportion of surgical aortic valve procedures performed by trainees in each of the three career stage: early (1^st^-2^nd^ year of training), mid (3^rd^-4^th^ year of training) and late (after 5^th^ year of training).


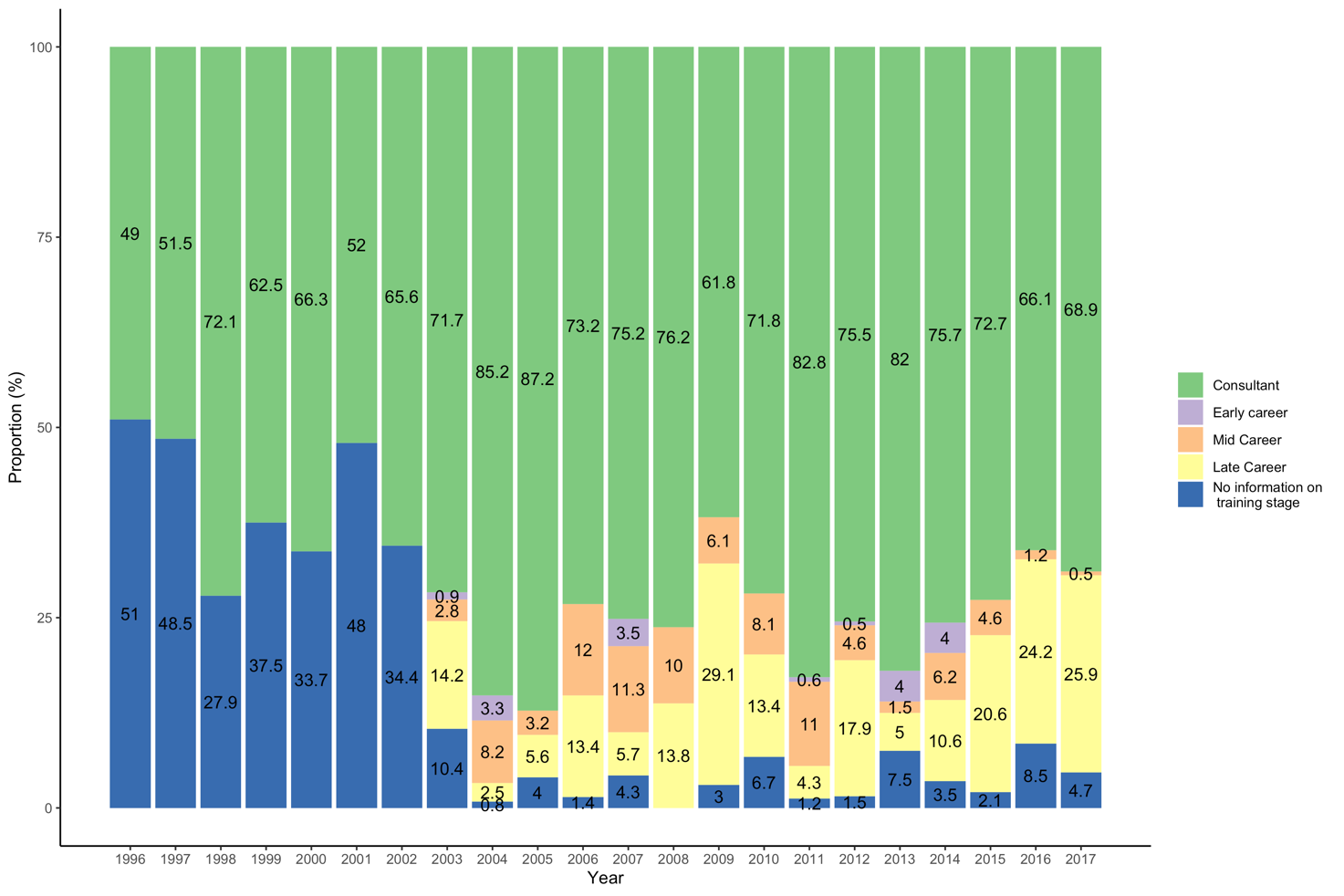


**Supplementary Figure 5.**  Love plot showing the changes in standardized mean difference before and after propensity score matching. It demonstrates that the balance of covariates was improved on all variables, which are below the threshold of 0.1 of absolute mean difference.


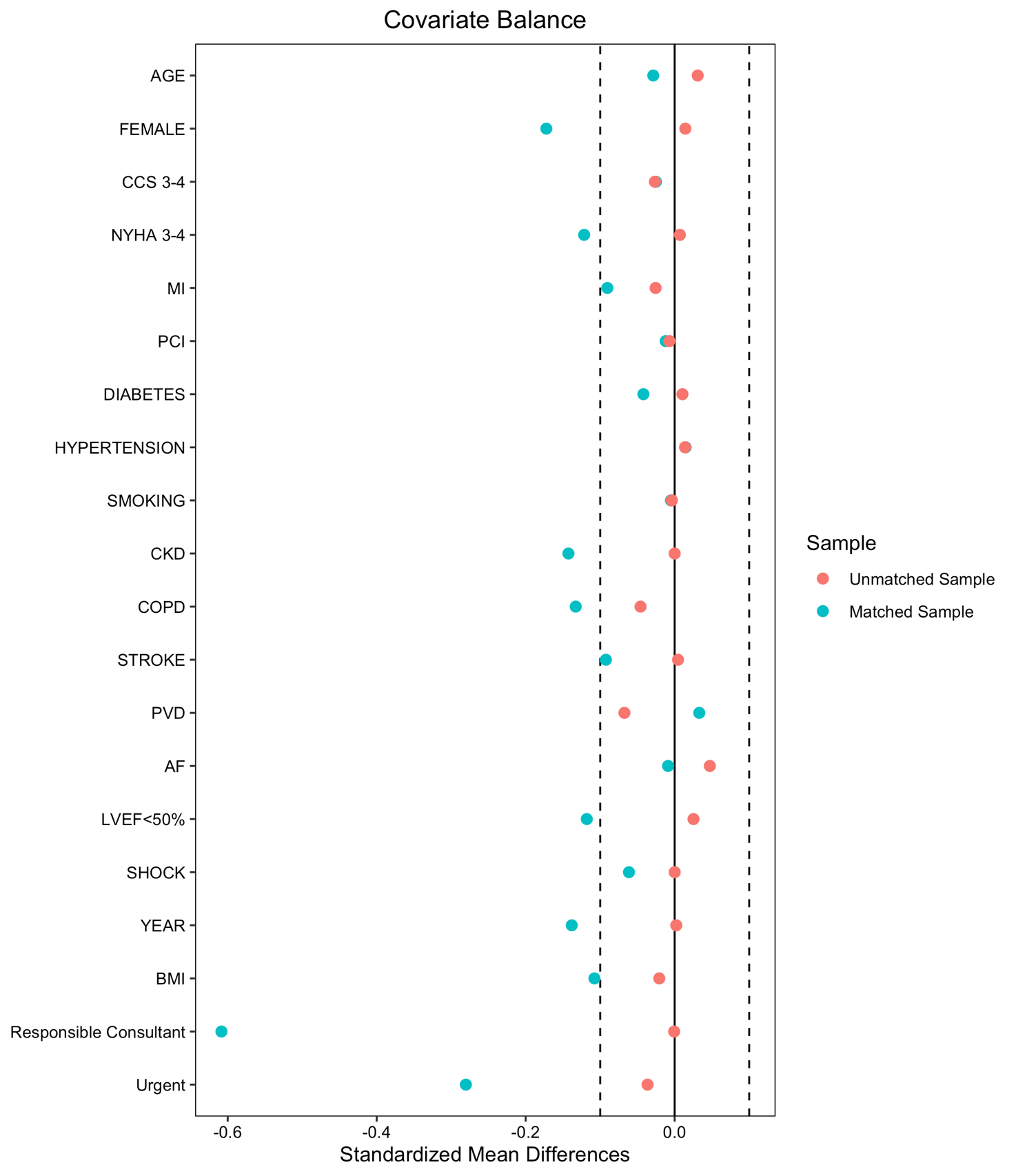


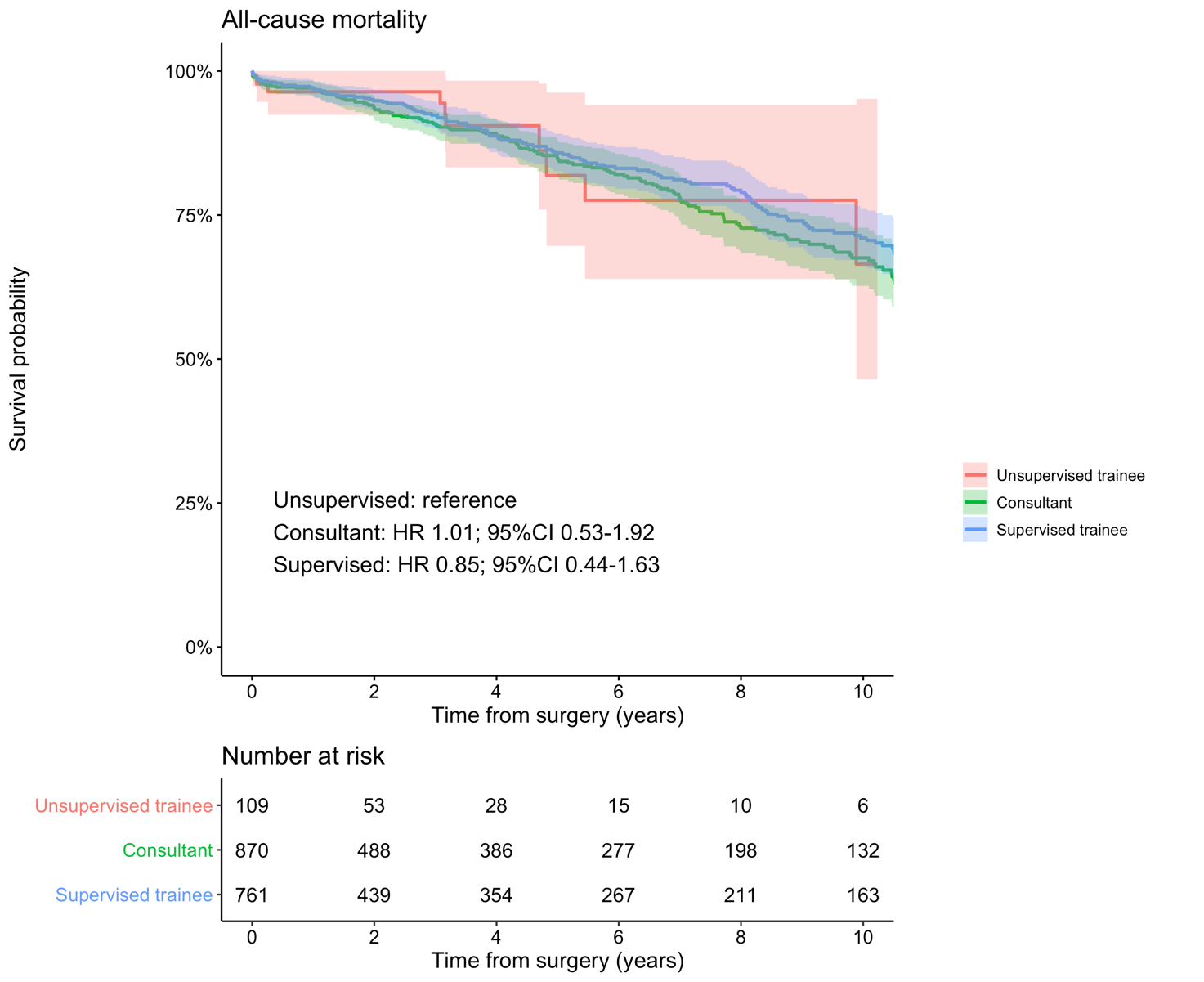
**Supplementary Figure 6.** Kaplan-Meier curves describing the cumulative survival probability in patients undergoing isolated surgical aortic valve replacement performed by consultants or unsupervised trainees.

**
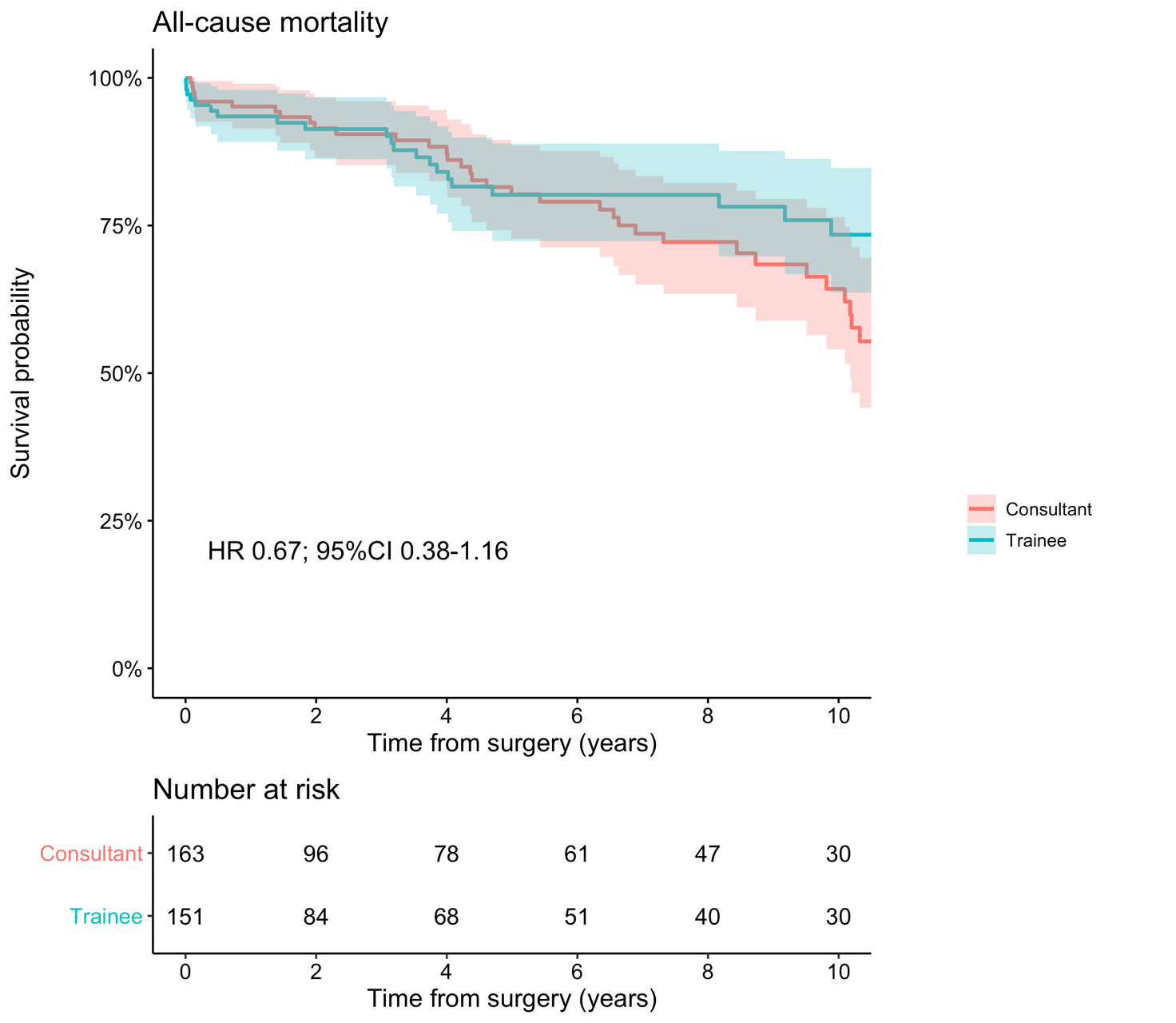
Supplementary Figure 7.** Kaplan-Meier curves describing the cumulative survival probability in patients undergoing urgent isolated surgical aortic valve replacement performed by consultants or trainee.
